# New Concepts in Diagnosis, Risk Factors and Work Ability in Patients with Hematogenous Spinal Infection

**DOI:** 10.3390/jcm11185470

**Published:** 2022-09-17

**Authors:** Panagiotis Korovessis

**Affiliations:** Chief Emeritus Orthopedic Department, General Hospital “Agios Andreas”, 262224 Patra, Greece; korovess@otenet.gr

In the last decades, there is an increasing incidence of hematogenous septic spinal infection (HIS). However, HIS appears with not specific symptomatology & findings on presentation that seem to lead to delay diagnosis and result in delayed start of appropriate therapy [1]. Regarding terminology and anatomical location of the infection, the affection of the disc is called spondylodiscitis (SSD) with the higher incidence of 95%; however, if the infection affects the vertebral endplates, it is called spondylitis or vertebral osteomyelitis (PVO). Most often, at the time of diagnosis establishment, the infection has infiltrated both the disc and vertebral body, thus, both terms are frequently used. HSI is a serious disease associated with an average mortality of 20% [2,3,4,5,6].

Due to the non-specific early clinical signs and symptoms, a significant delay, usually 2-6 months, has been reported between the first onset of symptoms and the establishment of a diagnosis and subsequent treatment. This considerable delay may lead to severe complications, such as neurologic deficit or even death, while the often-coexistent endocarditis may increase mortality. In this Editorial, we discuss a few recently published papers reporting on contemporary advanced diagnostic tools, risk factors, current treatment options, and therapeutic outcomes of HSI.

The contemporary principles in the diagnosis of HSI include MRI, repeated blood cultures, and percutaneous or intraoperative biopsy-culture. The mainstream in the treatment of HSI is the administration of antibiotics, bed rest and bracing, but if HSI is complicated (intractable spinal pain, neurologic impairment and/or abscess formation), surgical treatment is considered [3,7]. Previous studies have reported on the prognosis in patients with HSI (recurrence, mortality, functional outcome, and quality of life) [2,4,5,6,8,9]. Both spinal infection recurrence and neurological impairment may occur in one third of patients with HSI [10]. Thus, HSI often results in decreased physical function and disability, in addition to increased mortality [2,3,4,5,10,11,12].

Previous relative studies have discussed some risk factors for SSD. Heuer A et al. [13] retrospectively analyzed the charts from 307 patients with SSD and developed a so-called “risk assessment score”, using quickly available parameters to predict the “in-hospital mortality” of patients who were admitted suffering from SSD. These authors [13] identified specific clinical characteristics predictive of mortality for each individual patient admitted with SSD. This study reported that 14% of patients with SSD died during their hospitalization at a highly specialized center for Spine Surgery. More specifically, this paper showed that patients aged >72.5 years with rheumatoid arthritis, renal insufficiency (creatinine > 1.29 mg/dL) and CRP > 140.5 mg/L are at an increased risk of mortality by 3.9-times, 9.4-times, 4.3-times and 4.1-times, respectively. Staph. aureus, a causative agent, was shown to increase the risk of mortality by 2.3-fold. The authors concluded that their “Hamburg Spondylodiscitis Assessment Score” (HSAS) is able to precisely identify patients at a low-, moderate-, high- and very high risk of in-hospital mortality on admission. The limitations of this study include its single-center design, short term follow-up and predominantly Caucasian patients. However, the implementation of this novel prognostic method in the daily clinical practice of spine centers could, according to the authors, identify high-risk patients earlier.

CT-guided biopsy is the most commonly used method for the diagnosis of SSD in regard to causative pathogen detection. Alexander Braun A et al. [14] carried out an in-depth diagnosis and revealed the CT-imaging characteristics and clinical parameters for the successful prediction of microbiological pathogens in ambiguous SSD cases. Non-sclerotic endplate erosions in the CT scan and paravertebral/epidural abscess formation in MRI and CRP > 50 mg/L were evaluated by the authors for their predictive value. Endplate erosions appeared with a rate of 54.5% and 3.4% in patients with positive and negative culture, respectively. Paravertebral and/or epidural abscess, respectively, were present in 63.6% of patients with (+) culture and 24.1% in patients with (-) culture. CRP > 50 was reported in 63.6% and in 24.1% patients with (+) and (-) culture, respectively. It is impressive that three double combinations revealed high specificity (abscess and non-sclerotic endplate erosions, 100%; abscess plus CRP > 50, 96.6%; non-sclerotic endplate erosions and CRP > 50, 96.6%). The limitations of this study included its retrospective nature, small patient sample and single center design. However, the authors warned physicians that their study does not intend to inform clinical “decision making” on an individual basis. Therefore, the presented results should not be used to make a final judgment about performing or not performing CT-biopsies in SSD cases without further confirmatory validation studies.

Many cases of PVO associated with septic endocarditis have been reported, and physicians are warned to pay attention to the possibility of the coexistence of both diseases. However, the incidence, clinical presentation, and outcomes of endocarditis simultaneously with PVO remain unclear. Tadatsugu Morimoto et al. [15] studied all patients with PVO without a history of spinal surgery for endocarditis using Echocardiography and modified Duke criteria for endocarditis. Endocarditis was diagnosed in 12% of the cases with PVO. However, there are no significant differences in the clinical findings, causative pathogens, or imaging findings between simultaneous PVO and endocarditis infection and PVO without endocarditis patients. The main limitation of this study was the lack of specific clinical and laboratory findings that may prevent the diagnosis of IE. Therefore, spine surgeons and cardiologists involved in spinal infection treatment should be aware of co-existing endocarditis and should perform routine echocardiograms and repeated blood cultures, as early diagnosis and therapy are essential for minimizing morbidity and mortality.

Degenerative Modic type 1 (MT1) endplate changes and SSD frequently display similar findings. Ursula Schwarz-Nemec [16], using MRI, investigated vertebral bone marrow and endplate changes for their differentiation in patients with MT1, early SSD without abscess, and advanced SSD with abscess on sagittal unenhanced T1-weighted images. Endplate contours, extension of bone edema, and percentage ratios of T1-signal of MT1 (31.96%) were significantly less compared with early SSD (56.42%) and advanced SSD(91.84%). The highest diagnostic accuracy in identifying MT1 was provided by an “irregular, however intact endplate contour”. The authors indicate that this MRI feature enables safe differentiation between MT1 and SSD, particularly in the early stages of spinal infection. This study has several limitations, including its retrospective nature and small sample of patients, including only 19 early SSD cases; the biopsy results are also lacking in MT1 patients, where the diagnosis is based solely on clinical features, (-) blood cultures, and imaging findings. Additionally, this study does not shed light on the debated infectious pathogenesis of MT1, while information is lacking regarding potential pre-treatment with medication before being referred to the hospital.

Minimally invasive and percutaneous endoscopic surgery has become a popular treatment for spinal disorders; it reliably decreases open approach-related complications and comorbidity in the elderly and severely immune compromised patients. The advantages of percutaneous endoscopic debridement and pus drainage (PEDD) for PVO include direct observation of the lesion, direct pus drainage, and earlier pain relief. Tsai-Sheng Fu et al. [17], in a retrospective study, compared patients who underwent PEDD with those who underwent traditional anterior open debridement and interbody fusion with bone grafting. The causative agents, most commonly Staph. aureus, were isolated in a similar percentage of patients with PVO who received either FEDD or open surgery. However, in the PEDD group, blood loss was less than in open surgery. In the two-year follow-up radiographs, 86.7% of open surgery patients showed bony fusions of the infected segments. On the contrary, sclerotic change in the destructive vertebral endplates was observed and the motion of infected spinal segments was still preserved in the PEDD group. There was no significant difference in the change in sagittal profile, including primary correction gain, correction loss, and actual correction gain/loss. The limitations of this study were retrospective, not randomized, with selection bias by different surgeons and methods and a small patient sample.

Several studies, some recently published, have documented the safety and efficacy of early spinal instrumentation for PVO. However, very few of these studies have researched the recurrence rate or associated factors based on this specific group of patients. A retrospective cohort study by Jeong Seop Lim and Tae-Hwan Kimusing [18] investigated the recurrence rate and its associated factors in a large number of patients who underwent instrumented surgery for SSD within 6 weeks after diagnosis establishment. Only 10% of these patients showed recurrence. The recurrence rates in the authors cohort decreased from 14% to 8% at 2 to 6 weeks, respectively, in relation to the duration of antibiotic treatment for recurrence. The identified factors associated with recurrence were age >70 years, posterior approach in the thoracic spine, multiple surgical approaches, mesh cage, blood transfusion, antibiotics for resistant organisms, and systemic steroid administration over 2 weeks. There are several limitations associated this study. First, the database is a national claims database and was not originally designed for clinical research. Second, information related to possible risk factors such as precise surgical profiles (surgical protocols, and techniques among hospitals or surgeons) were not included in this study, and the data for causative organisms were substituted with those for the use of antibiotics for resistant organisms; this approach may have led to bias. Furthermore, the authors considered the administration of antibiotics efficient for spinal infection for 6 weeks according to the international guidelines for SSD; however, since spinal infections rarely persist after 6-week antibiotic regimens, this could have led to bias.

Very rarely, scientists have studied and reported the work status of patients treated for HSI. Yagdiran A et al. [19], in a fairly recent study, analyzed work status after treatment for HSI, as well as risk factors associated with loss of the patient’s ability to work (ATW). The authors used work status as their primary endpoint after one year and compared patients’ characteristics “at-work” versus “not-at work”. The present study, analyzing the workforce among a cohort of working-age HSI, patients was able to show that 42% of the patients did not return to work at the follow-up observation of one year after treatment. Patients working in a hard physical environment were no longer able to regain their pre-treatment work. The main factors associated with a sustained ATW included a low number of comorbidities (≤1) and a BMI >25. However, 27% received a disability pension one year after treatment. The authors recommended more support in retraining following successful treatment to maintain ATW and reduce the socio-economic burden. The limitations include selection bias, single-center study and the small sample of patients.

## References

[1] Nickerson E.K., Sinha R. (2016). Vertebral osteomyelitis in adults: An update. Br. Med. Bull..

[2] Kehrer M., Pedersen C., Jensen T.G., Hallas J., Lassen A.T. (2015). Increased short- and long-term mortality among patients with infectious spondylodiscitis compared with a reference population. Spine J..

[3] Herren C., Jung N., Pishnamaz M., Breuninger M., Siewe J., Sobottke R. (2017). Spondylodiscitis: Diagnosis and Treatment Options. Dtsch. Ärzteblatt Int..

[4] Aagaard T., Roed C., Dahl B., Obel N. (2016). Long-term prognosis and causes of death after spondylodiscitis: A Danish nationwide cohort study. Infect. Dis..

[5] Aagaard T., Roed C., Larsen A.R., Petersen A., Dahl B., Skinhøj P., Obel N., Danish Staphylococcal Bacteraemia Study Group (2014). Long-term mortality after Staphylococcus aureus spondylodiscitis: A Danish nationwide population-based cohort study. J. Infect..

[6] Yagdiran A., Otto-Lambertz C., Lingscheid K.M., Sircar K., Samel C., Scheyerer M.J., Zarghooni K., Eysel P., Sobottke R., Jung N. (2021). Quality of life and mortality after surgical treatment for vertebral osteomyelitis (VO): A prospective study. Eur. Spine J..

[7] Pola E., Taccari F., Autore G., Giovannenze F., Pambianco V., Cauda R., Maccauro G., Fantoni M. (2018). Multidisciplinary management of pyogenic spondylodiscitis: Epidemiological and clinical features, prognostic factors and long-term outcomes in 207 patients. Eur. Spine J..

[8] Gupta A., Kowalski T.J., Osmon D.R., Enzler M.J., Steckelberg J.M., Huddleston P., Nassr A., Mandrekar J.M., Berbari E.F. (2014). Long-Term Outcome of Pyogenic Vertebral Osteomyelitis: A Cohort Study of 260 Patients. Open Forum Infect. Dis..

[9] Sobottke R., Röllinghoff M., Zarghooni K., Zarghooni K., Schlüter-Brust K., Delank K.-S., Seifert H., Zweig T., Eysel P. (2009). Spondylodiscitis in the elderly patient: Clinical mid-term results and quality of life. Arch. Orthop. Trauma. Surg..

[10] Rutges J.P.H.J., Kempen D., Van Dijk M., Oner F.C. (2016). Outcome of conservative and surgical treatment of pyogenic spondylodiscitis: A systematic literature review. Eur. Spine J..

[11] Dragsted C., Aagaard T., Ohrt-Nissen S., Gehrchen M., Dahl B. (2017). Mortality and health-related quality of life in patients surgically treated for spondylodiscitis. J. Orthop. Surg..

[12] O’Daly B.J., Morris S.F., O’Rourke S.K. (2008). Long-term Functional Outcome in Pyogenic Spinal Infection. Spine.

[13] Heuer A., Strahl A., Viezens L., Koepke L.-G., Martin Stangenberg M., Marc Dreimann M. (2022). The Hamburg Spondylodiscitis Assessment Score (HSAS) for Immediate Evaluation of Mortality Risk on Hospital Admission. J. Clin. Med..

[14] Braun A., Germann T., Wünnemann F., Weber M.-A., Schiltenwolf M., Akbar M., Burkholder I., Kauczor H.-U., Rehnitz C. (2020). Impact of MRI, CT, and Clinical Characteristics on Microbial Pathogen Detection Using CT-Guided Biopsy for Suspected Spondylodiscitis. J. Clin. Med..

[15] Morimoto T., Hirata H., Otani K., Nakamura E., Miyakoshi N., Terashima Y., Wada K., Kobayashi T., Murayama M., Tsukamoto M. (2022). Vertebral Osteomyelitis and Infective Endocarditis Co-Infection. J. Clin. Med..

[16] Schwarz-Nemec U., Friedrich M.K., Stihsen C., Schwarz K.F., Trattnig S., Weber M., Josef G., Grohs G.J., Nemec F.S. (2020). Vertebral Bone Marrow and Endplate Assessment on MR Imaging for the Differentiation of Modic Type 1 Endplate Changes and Infectious Spondylodiscitis. J. Clin. Med..

[17] Fu T.-S., Wang Y.-C., Lin T.-Y., Chang C.-W., Wong C.-B., Su J.-Y. (2019). Comparison of Percutaneous Endoscopic Surgery and Traditional Anterior Open Surgery for Treating Lumbar Infectious Spondylitis. J. Clin. Med..

[18] Lim J.S., Kim T.H. (2022). Recurrence Rates and Its Associated Factors after Early Spinal Instrumentation for Pyogenic Spondylodiscitis: A Nationwide Cohort Study of 2148 Patients. J. Clin. Med..

[19] Yagdiran A., Bredow J., Weber C., Basha M.G., Eysel P., Fischer J., Jung N. (2022). The Burden of Vertebral Osteomyelitis—An Analysis of the Workforce before and after Treatment. J. Clin. Med..

